# Evaluation and Application of a Customizable Wireless Platform: A Body Sensor Network for Unobtrusive Gait Analysis in Everyday Life

**DOI:** 10.3390/s20247325

**Published:** 2020-12-20

**Authors:** Markus Lueken, Leo Mueller, Michel G. Decker, Cornelius Bollheimer, Steffen Leonhardt, Chuong Ngo

**Affiliations:** 1 Medical Information Technology, RWTH Aachen University, Pauwelsstr. 20, 52074 Aachen, Germany; leo.mueller@rwth-aachen.de (L.M.); michel.georges.decker@rwth-aachen.de (M.G.D.); leonhardt@hia.rwth-aachen.de (S.L.); ngo@hia.rwth-aachen.de (C.N.); 2 Department of Geriatrics, RWTH Aachen University Hospital, Pauwelsstr. 30, 52074 Aachen, Germany; cbollheimer@ukaachen.de

**Keywords:** body sensor network, gait analysis, inertial sensors, ground reaction force

## Abstract

Body sensor networks (BSNs) represent an important research tool for exploring novel diagnostic or therapeutic approaches. They allow for integrating different measurement techniques into body-worn sensors organized in a network structure. In 2011, the first Integrated Posture and Activity Network by MedIT Aachen (IPANEMA) was introduced. In this work, we present a recently developed platform for a wireless body sensor network with customizable applications based on a proprietary 868MHz communication interface. In particular, we present a sensor setup for gait analysis during everyday life monitoring. The arrangement consists of three identical inertial measurement sensors attached at the wrist, thigh, and chest. We additionally introduce a force-sensitive resistor integrated insole for measurement of ground reaction forces (GRFs), to enhance the assessment possibilities and generate ground truth data for inertial measurement sensors. Since the 868MHz is not strongly represented in existing BSN implementations, we validate the proposed system concerning an application in gait analysis and use this as a representative demonstration of realizability. Hence, there are three key aspects of this project. The system is evaluated with respect to (I) accurate timing, (II) received signal quality, and (III) measurement capabilities of the insole pressure nodes. In addition to the demonstration of feasibility, we achieved promising results regarding the extractions of gait parameters (stride detection accuracy: 99.6±0.8%, Root-Mean-Square Deviation (RMSE) of mean stride time: 5ms, RMSE of percentage stance time: 2.3%). Conclusion: With the satisfactory technical performance in laboratory and application environment and the convincing accuracy of the gait parameter extraction, the presented system offers a solid basis for a gait monitoring system in everyday life.

## 1. Introduction

Healthcare costs have risen massively in recent years and the trend does not seem to be reversed. According to the United States (US) National Health Expenditure, spending has tripled in the US since 1995 and amounted to 18% of GDP in 2018 [1]. With an increasing life expectancy together with a stagnating birth rate in the corresponding countries, an aging society is being observed. The actual effects of this development are difficult to estimate and are the subject of current studies. It can be assumed that the burden on health care systems will continue to increase. Therefore, new cost-effective concepts for medical care are needed to maintain the standard of living and the quality of care.

Body sensor network (BSN), alternatively wireless body area network (WBAN), technology could make a decisive contribution to relieving the burden on health systems and thus keeping them viable for society in the future [2,3]. A (wireless) body sensor network as defined in the IEEE 802.15.6 standard is a specific wireless network realization that combines body-worn sensors of a single (human or animal) being into a dedicated network. By definition, this makes the most important difference to wearables in the conventional sense or single body-worn sensors, such as a Holter ECG. BSNs, which in general are not strictly limited to wireless communication, have originally been introduced for data acquisition in personalized healthcare. Portable measurement systems can facilitate patient monitoring and alert healthcare professionals in emergencies such as heart attacks or falls. The use of these systems in everyday life could provide access to data that were previously difficult to be obtained and could offer new therapeutic approaches [4]. These inconspicuous interventions could lead to immense financial long-term savings and a considerable improvement in the quality of life. To increase the acceptance of the devices, the sensors must not become a burden for their wearers. In this sense, unobtrusiveness applies to many different aspects, such as wearing location, battery charging, handling for both patients and medical staff, and many others.

The choice of the radio–communication interface has to be carefully considered during the development process since a variety of different communication standards is eligible for the data transfer within a BSN. The carrier-frequency is directly connected to the respective communication standard (Wi-Fi: 2.4GHz/5GHz, Bluetooth: 2.4GHz, ZigBee: 900MHz/2.4GHz). As stated in previous studies and theoretical considerations [5,6,7], lower frequency bands have lower signal attenuation as compared to the 2.4GHz band. The Industrial, Scientific, and Medical (ISM) band and the Short Range Device (SRD) band provide additional frequency bands at 433MHz and 868/915MHz, respectively. These frequency bands allow for the implementation of an application-specific proprietary protocol under certain restrictions. Proprietary communication standards thus can avoid commonly known complications such as collisions with similar communication standards in the 2.4GHz band, data rate limitations, high power consumption, or shadowing effects of human tissue towards higher frequencies. Especially, the latter has to be considered for a BSN application for gait analysis.

Many research projects have addressed mobile sensorized monitoring systems for varying health and fitness applications. Specifically, in the area of gait analysis, three sensor modalities have proven high usability for wearable monitoring systems: inertial measurement units (IMUs) including magnetic field sensors to monitor posture and motion, insole-integrated force sensing resistors to monitor ground reaction forces (GRFs), and surface-mounted electromyographic (sEMG) sensors to monitor muscular activity [8,9,10,11,12]. The latter is not considered for long-term monitoring due to the use of electrodes that have to be attached to the patient’s skin. In contrast, the IMU and GRF sensors are extensively integrated into scientific research platforms in long-term monitoring applications for gait analysis [8,12].

IMUs, which include accelerometers and gyroscopes, were first proposed as a functional tool for gait analysis half a century ago [13] and has gained importance with the development of low-cost piezo-resistive and micro-electromechanical inertial sensors [14]. In recent years, IMUs were integrated into different mobile motion and activity monitoring devices [8,15,16,17,18,19,20,21,22,23]. These devices usually consist of one single node [23,24] or multiple sensing nodes that are organized in a BSN [8,18,20]. For both single and multiple node applications, different sensor locations were proposed and investigated for different types of targeting diseases.

GRF sensing devices usually consist of different kinds of pressure sensors that are attached to the insole or the shoe of the subject. Pressure sensors are implemented by commercially available sensors, such as force sensing resistors (FSRs) [25,26,27,28,29,30], flex sensors [29] and barometric pressure sensors [31], or customized solutions, such as electronic textiles-based pressure sensors [32,33], light-sensing chambers with pressure-actuated optical barriers [34] or carbon-embedded piezo-resistive materials [35].

**Figure 1:**
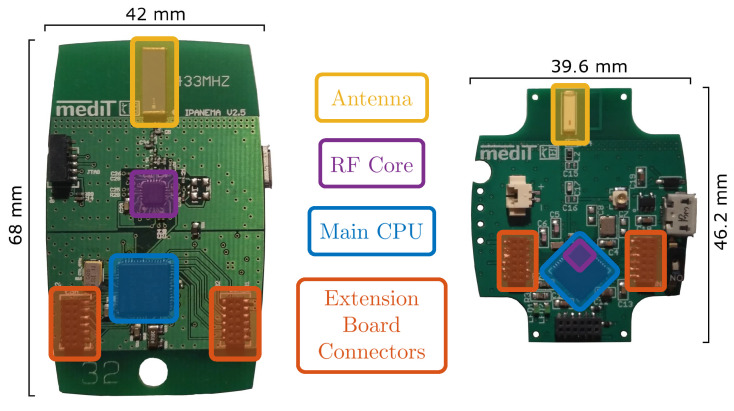
Comparison of the board layouts of the Integrated Posture and Activity Network by MedIT Aachen (IPANEMA) v2.5 (**left**) and v3.0 mainboards (**right**). The most important components are color-coded for each board.

In this work, a modular BSN for long-term motion monitoring is designed and tested. It follows the basic ideas of the Integrated Posture and Activity Network by MedIT Aachen (IPANEMA). The first version of IPANEMA was developed in 2007 [36]. In 2009 and 2011, the BSN was extended by version 2.0 and 2.5, respectively [37]. The third version was recently finalized with a completely revised design. A comparison of the IPANEMA v2.5 mainboard to the mainboard of IPANEMA v3.0 is shown in Figure 1. Table 1 additionally compares the main features of both versions. As an application scenario of the recently developed platform, we present a sensor configuration for unobtrusive gait analysis within this contribution. The sensor network consists of three identical IMU sensors and a single FSR-based insole for recording of GRFs. Since the IMU-based evaluation for this study was already presented in previous publications [38,39], this specific contribution focuses on the evaluation of the technical reliability of the whole system and the extension by an FSR-based GRF sensor.

## 2. Methods and Material

### 2.1. The IPANEMA Mainboard

A single network node of a BSN basically contains a CPU, a radio unit (i.e., transceiver and antenna), an energy management system, and specific measurement circuits for the acquisition of physiological or biomechanical parameters. In the case of IPANEMA, each node is equipped with a mainboard, a sensor board, and a 230mAh lithium-ion battery. The mainboard contains the CPU, the RF transceiver and an antenna, the power supply unit, and a sensor controller (SC) that directly connects to the sensor boards via two socket-connectors (compare (cf.) Figure 1). The key component of the IPANEMA v3.0 mainboard is the CC1310 sub-1GHz wireless micro-controller (Texas Instruments). It contains an ARM Cortex-M3 main CPU, an RF controller, an integrated sensor controller, and several peripherals such as a Real-Time Clock (RTC) module, an AES-128 encryption module, a Direct Memory Access (DMA) controller, different communication interfaces, and timer modules. The RF controller is based on an ARM Cortex-M0 architecture. It supports several sub-1GHz ISM and SRD bands such as the 433 MHz, 868 MHz, and 915 MHz. In contrast to IPANEMA v2.5, the physical layer of v3.0 is based on the 868/915 MHz band. To overcome communication conflicts, European regulations prescribe channel sensing for duty cycle-free occupancy of these sub-bands [40]. In the IPANEMA v3.0 protocol stack, Polite Spectrum Access (PSA) is implemented by a Listen-Before-Talk (LBT) approach, where the current Receive Signal Strength Indicator (RSSI) is used to determine the channel occupancy.

The basic idea of the IPANEMA BSN is to serve as a flexible scientific communication platform for different sensor modalities. Therefore, the mainboard contains expansion ports to connect to different types of sensor boards. The connectors contain the 3.3V supply and battery voltage, and additional pins that directly connect to the multi-functional general-purpose input/output (GPIO) pins of the integrated SC. The SC operates completely independent from the main CPU and organizes the communication with the respective sensor board, i.e., the initialization and configuration process, reading data from analog or digital sensors, and triggering the CPU.

**Figure 2:**
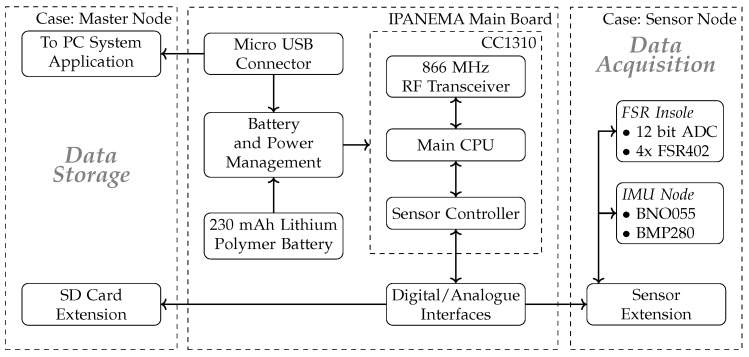
The IPANEMA mainboard can be equipped with either the master node extension board or a specific sensor board. The master board contains a USB port that establishes the data connection to the corresponding PC application. The sensor board is equipped with different types of sensors depending on the target modality.

For communicating with the sensor boards, the SC provides different standard interfaces such as the Inter-Integrated Circuit ($I^{2}C$) bus, the Inter-IC Sound ($I^{2}S$) bus, the Serial Peripheral Interface (SPI), and the Universal Asynchronous Receiver Transmitter (UART) bus. It also contains an integrated 12-bit Analog-to-Digital Converter (ADC), several interrupt functions, comparators, and counting modules to interact with measurement circuits. The sampling rate can be adapted individually for each different sensor modality within the SC configuration. The SC and the main CPU share a dedicated memory space to exchange data to be further processed and sent over the network. The main CPU organizes the communication following the proprietary network protocol. Sensor data are retrieved from the shared memory and transmitted in network packets. The data are received by the master node and either forwarded to a PC system via a USB port or stored on an integrated micro SD card for long-term monitoring purposes. For telehealth applications, the data are additionally transferred to large-scale memory servers for further processing the obtained data. A graphical overview of the system is given in Figure 2.

### 2.2. Proprietary Network Protocol

**Figure 3:**
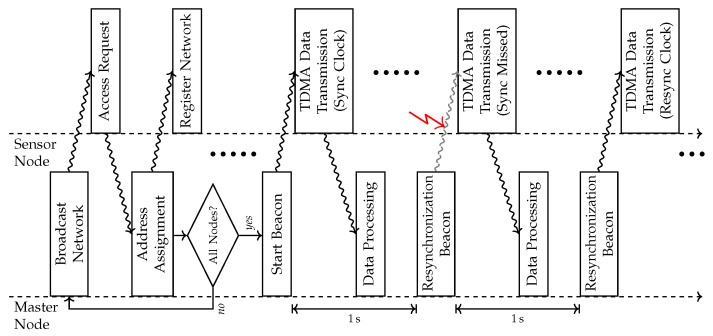
Timing diagram according to the communication procedure between the master node (MN) and sensor node (SN) including initialization routine and standard data acquisition procedure.

The IPANEMA BSN consists of a master node (MN) and several sensor nodes (SNs) that are organized in a star-shaped network. The proprietary internal network protocol is based on this topology. The MN initializes the network structure for a predefined maximum number of SNs connecting to the MN. The communication is controlled by a time division multiple access (TDMA) process that assigns dedicated time slots to each SN connected. A higher-level communication frame allows for periodical re-synchronization of the SNs and network error handling by the MN. For this purpose, the MN sends re-synchronization beacons each second and eventually reconnects lost SNs. In this case, a careful re-synchronization is not only necessary to comply with the dedicated time slots of the TDMA protocol, but additionally, ensure an accurate time-stamping of a single sampling point for any sensing modality. After each SN has registered and received its individual unique network ID, the MN broadcasts the start beacon initiating the TDMA protocol on all nodes (Figure 3).

The core communication and sampling procedures take into account different timing modules to maintain reliably identical clocks. These include the MN core clock module, the SN core clock module, and the RF clock module of the respective SN. The MN clock provides the internal global network time ${\left.T_{\mathrm{MN}}\right|}_{\mathrm{sync}}$ broadcasted within each re-synchronization beacon with a range of 32bit and a resolution of $\Delta T_{\mathrm{LSB}}=10\;\mu s$. The RF module of each slave node captures the exact receiving time ${\left.T_{\mathrm{RF},\mathrm{SN}}\right|}_{\mathrm{sync}}$ of the re-synchronization packet from the isolated RF clock. When a packet is received by the RF module, it triggers an event for further processing. The temporal delay between receiving and processing the packet is obtained from the RF clock module

(1) $$\Delta T_{\mathrm{proc}}={\left.T_{\mathrm{RF},\mathrm{SN}}\right|}_{\mathrm{proc}}-{\left.T_{\mathrm{RF},\mathrm{SN}}\right|}_{\mathrm{sync}}\;$$

where the current sensor timestamp ${\left.T_{\mathrm{SN}}\right|}_{\mathrm{sync}}$ and the processing timestamp ${\left.T_{\mathrm{RF},\mathrm{SN}}\right|}_{\mathrm{proc}}$ are obtained simultaneously from the local sensor clock module and the RF clock module, respectively. This way, each SN can calculate the exact sampling time with respect to the unique global network clock ${}^{\mathrm{MN}}T_{\mathrm{SN}}$

(2) $${}^{\mathrm{MN}}T_{\mathrm{SN}}\left[k\right]={\left.T_{\mathrm{MN}}\right|}_{\mathrm{sync}}+\left(T_{\mathrm{SN}}\left[k\right]-\left({\left.T_{\mathrm{SN}}\right|}_{\mathrm{sync}}-\Delta T_{\mathrm{proc}}\right)\right)$$

After receiving a synchronization beacon, the corresponding timestamps, ${\left.T_{\mathrm{MN}}\right|}_{\mathrm{sync}}$ and ${\left.T_{\mathrm{SN}}\right|}_{\mathrm{sync}}$, and the processing time $\Delta T_{\mathrm{proc}}$ are updated accordingly. If a synchronization beacon is missed, e.g., due to transmission errors, the previous timestamps are retained for further calculation of the current global network time until the next synchronization frame is received. This way, possible deviations of the oscillator components are compensated and both the TDMA protocol timing and the sampling clock are robust against hardware-dependent variances.

The internal 32-bit timer together with the timer resolution $\Delta T_{\mathrm{LSB}}$ allows for a maximum recording time $T_{\max}$ of

(3) $$T_{\max}=\frac{2^{32}\cdot 10\;\mu s}{3600}=11.93\;h,$$

which is in the range of the theoretical maximum operating time due to limitations of the battery capacity (cf. Table 1). Still, the network provides a comparably fine sampling resolution of 10μs. The integrated oscillator (TSX-3225 24MHz, Seiko Epson Corporation, Suwa, Japan) has a frequency tolerance of $f_{\mathrm{tol}}=\pm 10\;\mathrm{ppm}$. This results in a theoretical temporal deviation $\Delta T_{\mathrm{err},\max}$ according to component tolerances of

(4) $$\Delta T_{\mathrm{err},\max}=2^{32}\cdot 10\;\mu s\cdot \left(\pm 10\;\mathrm{ppm}\right)=\pm 0.43\;s.$$

In the context of the intended application of long-term gait analysis, this means a theoretical deviation of about one stride over 12h, if only the compensation of the static time offset is considered.

### 2.3. Inertial Measurement Node

The inertial measurement node consists of the integrated inertial measurement unit (IMU) BNO055 (Bosch Sensortec, Reutlingen, Germany) and an absolute barometric pressure sensor BMP280 (Bosch Sensortec, Reutlingen, Germany). The IMU contains an accelerometer, a gyroscope, a geomagnetic field sensor, and a Cortex M0+ microcontroller for on-chip sensor fusion. However, the magnetic field sensor was not used in our approach to reliable indoor orientation estimation. On the one hand, the usage of only accelerometer and gyroscope saves energy with respect to long-term monitoring applications. On the other hand, magnetic field sensors are often distorted by ferrous material or power lines within an indoor environment [41]. The accelerometer and gyroscope data were fused to obtain the current orientation of a sensor node. The barometric pressure sensor (BPS) includes a piezo-resistive sensing element and a temperature sensor to compensate for temperature-depending drifts. The BPS was utilized to estimate changes in absolute altitude for detection of stairs walking, floor elevating, sit-to-stand transitions, or drops. The communication was realized via the I${}^{2}$C bus for both integrated circuits. An overview of the characteristic parameters for the integrated sensors is given in Table 2.

### 2.4. Measurement of Ground Reaction Forces

**Figure 4:**
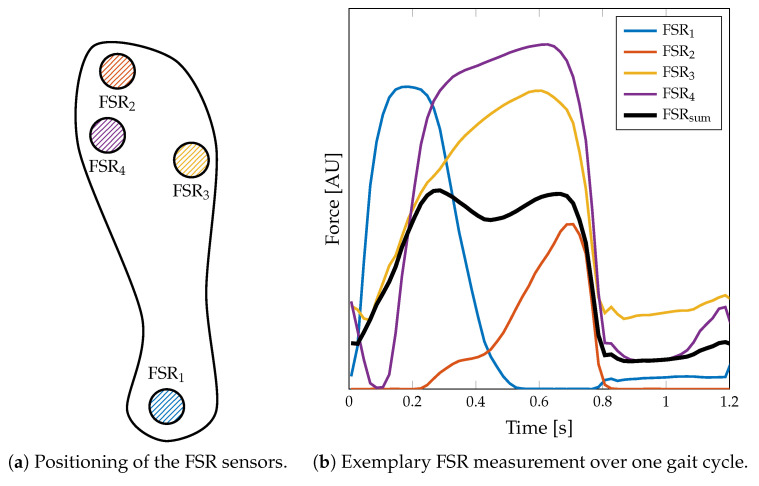
Implementation of the force sensing resistor (FSR) insole. (**a**) The insole is equipped with four FSR402 sensor pads located at the distal phalanges (${\mathrm{FSR}}_{2}$), the first (${\mathrm{FSR}}_{4}$) and the fifth metatarsophalangeal joints (${\mathrm{FSR}}_{3}$), and the heel (${\mathrm{FSR}}_{1}$). (**b**) An exemplary measurement of a step with this sensor setup. Note that the ${\mathrm{FSR}}_{\mathrm{sum}}$ does not reflect the actual force, since the setup is not calibrated to the individual pressure distribution.

The measurement of ground reaction forces (GRF) was implemented by integrating four force sensing resistors (FSRs) into an insole. The four FSR402 sensors (Interlink Electronics, Irvine, CA, USA) with a diameter of 13mm of sensing region were placed below (a) the distal phalanges (i.e., the big toe), (b) the first and (c) the fifth metatarsophalangeal joints, and (d) the heel (Figure 4a). The FSR sensors are subjected to a varying load in the successive phases of one gait cycle, as depicted in Figure 4b. This way, the GRF is sampled spatially along the different regions of the foot that mainly contribute to the overall GRF. The quantitative GRF approximation is determined by calculating the average (arithmetic mean) of the digital FSR signal components

(5) $${\mathrm{FSR}}_{\mathrm{sum}}\left[k\right]=\frac{1}{4}\sum_{i=1}^{4}{\mathrm{FSR}}_{i}\left[k\right].$$

**Figure 5:**
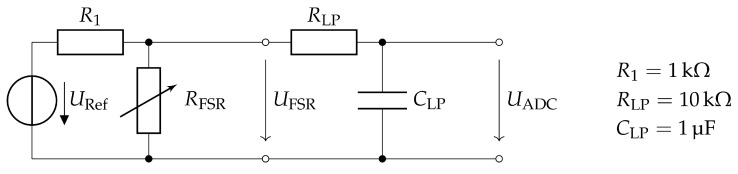
Hardware design of the FSR measurement circuit. Each FSR sensor is low-pass filtered to eliminate measurement noise and adapt to the gait dynamics.

Each FSR sensor is part of a voltage divider supplied by the reference voltage $U_{Ref}=3.3\;V$ (Figure 5). The resulting signal is filtered by a low-pass circuit with a corner frequency of $f_{c}=15.92\;\mathrm{Hz}$ and subsequently sampled by the 12-bit Analog-to-Digital Converter (ADC) MCP3204 (Microchip Technology Inc.). The ADC is connected to the sensor controller via the SPI bus.

### 2.5. Unobtrusive Sensor Setup for Gait Monitoring

While stationary gait analysis systems are traditionally used in movement laboratories, long-term monitoring systems require a maximized level of acceptance and compliance by the patient. Unobtrusive measurement of physiological or biomechanical parameters has become increasingly important in recent decades, as it provides insights into the everyday-life behavior of the patient. An unobtrusive sensor setup in a BSN application for gait analysis is characterized by wearable sensors that are located on the human body so that they do not affect natural motion and the patient ideally does not take note of the additional sensor equipment.

**Figure 6:**
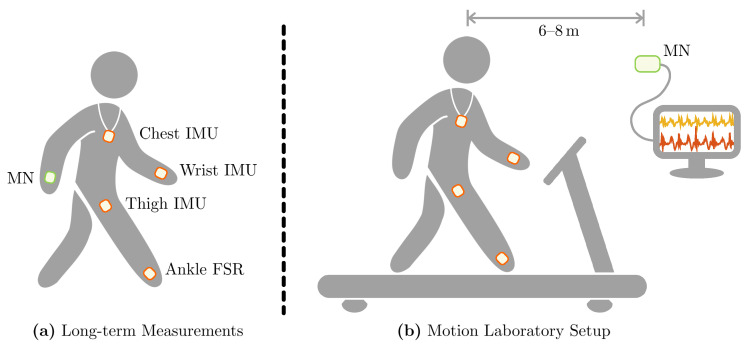
Application scenarios of the proposed sensor system realized in within this contribution. (**a**) The typical unobtrusive sensor setup for long-term measurements in everyday life. (**b**) Measurement setup during treadmill walking trials in the motion laboratory conducted for the validation process.

Smart electronic devices, i.e., mobile phones and smart-watches, and their integrated sensor units offer a promising opportunity to unobtrusively collect motion data. Concerning the fundamental idea of an unobtrusive sensor setup for everyday-life monitoring, we chose a setup including three IMU sensors, attached to the thigh, the wrist, and the chest as well as one FSR insole. This setup is motivated by typical wearable devices and their preferable wearing location: A cell phone in the pocket (thigh) and a smart-watch at the wrist supplemented by a pendant sensor for fall detection (cf. Philips Lifeline AutoAlert or similar) and an ordinary insole. With this sensor setup, we ensure on the one hand a broad acceptance for wearing these sensors and on the other hand cover movements of the lower extremities, the trunk, the upper extremities, and the ground contact. The typical applications are twofold. On the one hand, it is possible to conduct long-term measurements with the MN directly attached to the human body, in our case at the second wrist. On the other hand, the BSN can be used to record data in a motion laboratory environment, where the MN is connected to a PC system to enable online tracking of the data streams. The two application scenarios are depicted in Figure 6 showing the individual sensor locations for each case. Please note that in the motion laboratory the distance between MN and the participant walking on the treadmill was approximately 6–8 m due to infrastructural constraints.

### 2.6. Detection of Physical Activity in Long-Term Measurements

Detection of physical activity, more specifically the extraction of gait phases, during long-term measurements of everyday life activities is a key feature of the proposed sensor system. We identified the sensor node located at the thigh as the most reliable node for determining gait phases in specific. We implemented a simple algorithm to detect phases of physical activity using the signal vector magnitude (SVM) of the three-dimensional thigh acceleration data $a_{\mathrm{thigh}}={\left[a_{x}\;a_{y}\;a_{z}\right]}^{T}$

(6) $$\begin{array}{cc} \mathrm{SVM}[k] & ={\left||\;a\left[k\right]\;|\right|}_{2} \end{array}$$

(7) $$\begin{array}{cc} \; & =\sqrt{a_{x}^{2}\left[k\right]+a_{y}^{2}\left[k\right]+a_{z}^{2}\left[k\right]}. \end{array}$$

The standard deviation of the SVM, $\sigma_{\mathrm{SVM}}\left[i\right]$, was calculated window-based in the first step with a fixed window size of 1s. The SVM was binary sampled with a threshold of 0.1g,

(8) $$\begin{array}{c} \sigma_{\mathrm{SVM},\mathrm{bin}}\left[i\right]=\left\{\begin{array}{cc} 1, & \sigma_{\mathrm{SVM}}\left[i\right]\geq 0.1\;g\\ 0, & else \end{array}\right., \end{array}$$

which suppresses low-energy artifacts, such as low dynamic movements, and the global signal noise of the accelerometer of approximately 1.5mg. The resulting decision vector was median-filtered with a filter length of five samples, which equals a time section of five seconds. The averaged decision vector was finally filtered with a 1-dimensional opening function to eliminate short periods of physical activity, which for some reason may not be significant for further analysis. Please note that based on the algorithm, it is not possible to differentiate between any kind of physical activity, where the leg or thigh is majorly involved. Since every long-term recording associated with this contribution took place in the office environment and none of the participants reported differently, physical activity in this particular case is only referred to as walking activity.

### 2.7. Algorithm for Gait Segmentation Based on FSR Measurements

**Figure 7:**
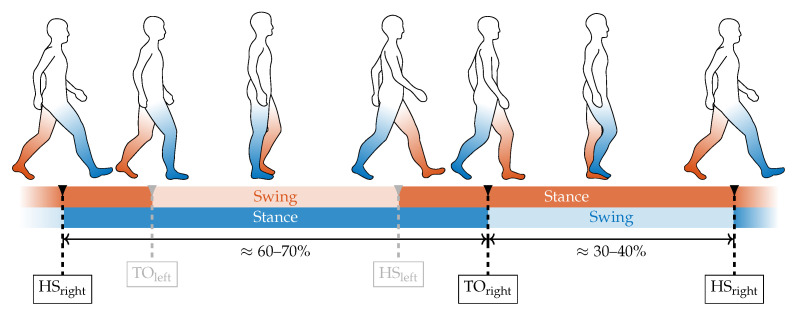
The two main components of the basic gait cycle consist of the stance phase and the swing phase. During normal gait, the stance phases of both left and right leg overlap during the double support phase. The stance and swing phases are separated by the heel strike (HS) and toe off (TO) event, which are the key characteristic points to be extracted from the GRF.

The most important characteristic points for wearable-based gait analysis are the heel strike (HS) and toe off (TO) events, respectively. These events indicate the two main phases in human gait, the stance phase, and the swing phase (Figure 7) and usually represent prominent and, therefore, easily detectable landmarks in wearable sensor data. Therefore, they are commonly used as key features in gait segmentation. Based on the specific application, other key features such as mid swing or mid stance can also be detected in wearable-based data. Since the FSR-based gait segmentation is in the main scope of this contribution we focus on the detection of HS and TO events. The summed-up FSR time series serves as the input signal for the segmentation algorithm. Since insole-integrated FSR measurements besides GRF are subject to additional motion artifacts due to contact pressure of the shoe and, therefore, differ from standard GRF measurements (i.e., force plate), we implemented a specific algorithm for HS and TO detection in FSR data.

**Figure 8:**
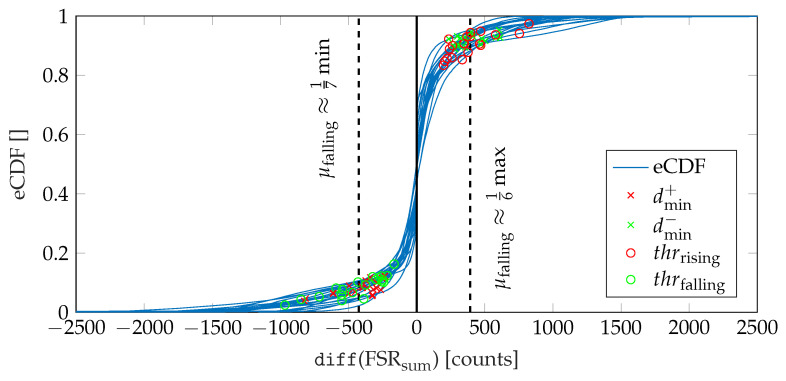
Illustration of the procedure for determining the threshold values used for the detection of HS and TO events.

Firstly, we calculate the local minimum of the summed-up FSR signal, which corresponds to the lowest pressure during one gait cycle. Since in higher walking speeds the plateau of the maximum foot pressure alters to a double peak due to the swing phase initiation of the opposite leg (cf. Figure 4b), the maximum peak pressure is not a reliable indicator. We assume a minimum distance of 0.7s between two adjacent peaks for the parameterization of the peak detection. Then, we identify the beginning of the falling (TO) and the rising edge (HS), respectively, starting from the local minimum. Therefore, we find the closest positive and negative value exceeding certain threshold values in the approximated derivative (diff()) of the summed-up FSR signal. These thresholds values were determined based on the FSR measurements conducted during the walking trials related to this contribution. Therefore, we investigated the empirical cumulative distribution function (eCDF) of the single difference signals of the summed-up FSR data. As shown in Figure 8, the eCDF can be separated in three main parts: a flat distribution region of negative signal slope, a relatively small transition region, and a flat region indicating positive slopes. This characteristic is mainly due to the signal shape of a band-limited rectangular signal. We identified the key characteristic points of the eCDFs, which we defined as the turning points from negative slope flat since these points statistically separate falling and rising edges from signal plateaus. For every single normalized eCDF, we identified the turning points by searching the minimum distance between the eCDF curve and [0,1] for $d_{\min}^{+}$ and [0,0] for $d_{\min}^{-}$, respectively. Subsequently, we calculated the mean value of the normalized amplitudes to define a fixed threshold with respect to the maximum and minimum amplitude values of each eCDF. Due to computational reasons we rounded the values to integer fractions.

Since the falling edge was found to be steeper as compared to the rising edge, the thresholds were identified as

(9) $$\begin{array}{cc} thr_{\mathrm{rising}} & =\frac{1}{6}\;\max_{k}\;\mathrm{diff}\left({\mathrm{FSR}}_{\mathrm{sum}}\right)\left[k\right]\;\mathrm{and} \end{array}$$

(10) $$\begin{array}{cc} thr_{\mathrm{falling}} & =\frac{1}{7}\;\min_{k}\;\mathrm{diff}\left({\mathrm{FSR}}_{\mathrm{sum}}\right)\left[k\right]. \end{array}$$

**Figure 9:**
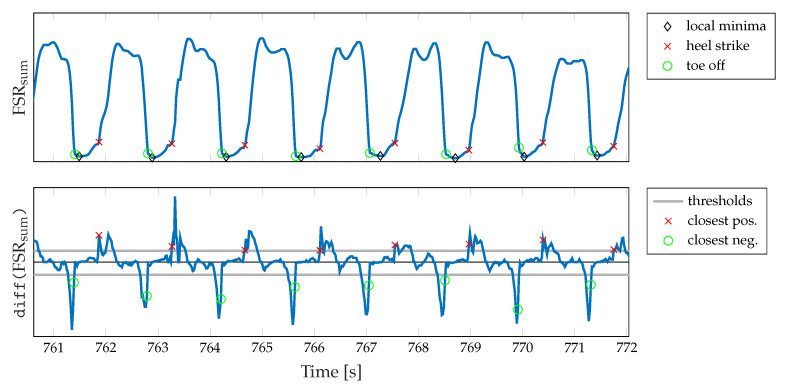
Exemplary illustration of the segmentation algorithm. The algorithm utilizes the summed-up FSR signal and its derivative, respectively, to identify TO and HS events during gait phases.

The basic procedure of identifying the threshold values is depicted in Figure 8. In addition, the final threshold values $thr_{\mathrm{rising}}$ and $thr_{\mathrm{falling}}$ according to Equations (9) and (10) are given. Finally, an exemplary illustration of the FSR-based gait segmentation algorithm is shown in Figure 9.

### 2.8. Trial Designs for the Validation of the Sensor Setup

Three different test scenarios were chosen to validate the performance of the proposed sensor setup. Firstly, the functional capability of the system was investigated in a technical validation setup in terms of transmission performance and timings. Secondly, we recorded long-term motion data during everyday life activities to validate the system reliability in a real-life application scenario. Finally, we conducted a smaller clinical study with ten participants to validate the activity detection algorithm and confirm the ability to correctly extract gait parameters from the obtained sensor data, respectively.

- Technical laboratory setup: The validation of the technical functionality in terms of transmission and timing performance was performed in a technical laboratory. The static measurements were conducted inside a laboratory room with as low electromagnetic interference as possible. The spatial signal strength measurements were conducted outside the building to reduce reflection disturbances. The SN was mounted on a turnable robot arm (Mitsubishi Movemaster) to avoid human tissue interference during the measurements and guarantee accurate spatial recordings.
- Long-term measurements: We conducted several long-term measurements with healthy subjects equipped with the proposed system during their everyday office life, while normally continuing their business. The records were used to validate the system reliability in a real-life scenario. Comparable to the technical validation, we focused on the evaluation of the transmission performance of the system.
- Gait parameter study: Since gait analysis is in the main scope of this contribution and the main purpose of the proposed system, we validated the system with respect to the extraction of gait parameters. We conducted a clinical trial in the movement laboratory of the Department of Geriatrics of RWTH Aachen University Hospital to compare the outcome of the system to clinically relevant gait parameters. The study was approved by the ethical committee of the University Hospital at RWTH Aachen University (Ref. No. EK 024-20) and included ten healthy, young participants (29.8±4.24years, 3 female, 7 male, average body height 175.7±8.65cm). In each trial, the participant was asked to walk on a treadmill (Zebris FDM-T, Zebris Medical GmbH) at different walking speeds (1–4 $\mathrm{km}\;h^{-1}$). The treadmill includes integrated force plates and generates the reference data for the gait parameters.

## 3. Results

### 3.1. Transmission Performance

The transmission performance of a wireless communication system can be quantified by two main characteristics: the Receive Signal Strength Indicator (RSSI) and the Packet Error Rate (PER). Several measurements were conducted recording the RSSI and the number of transmitted packets (NoP) for each SN under different environmental conditions to evaluate the transmission performance of the IPANEMA v3.0 mainboard. The PER can directly be calculated from the NoP. The performance tests were conducted in a technical laboratory setup, as well as during long-term measurements of everyday-life activities and gait trials in a movement laboratory. The RSSI and the NoP are automatically captured by the MN for each packet arriving.

**Figure 10:**
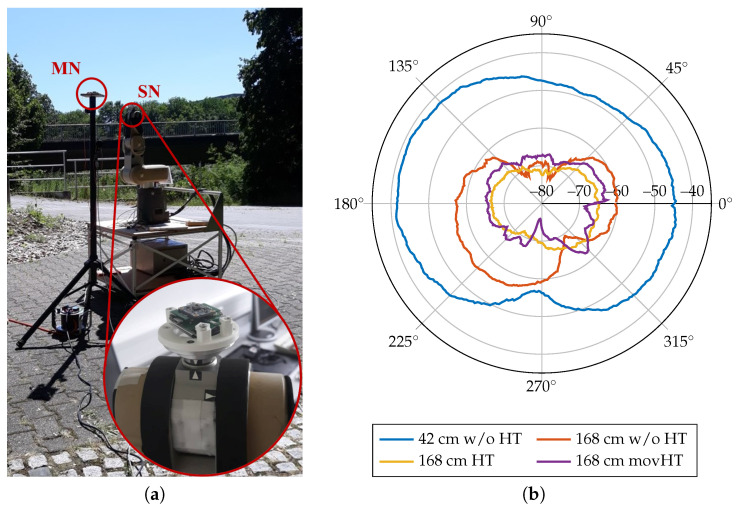
Receive Signal Strength Indicator (RSSI) measurements. (**a**) Measurement setup of the spatial RSSI recordings. The sensor node is placed at the end-effector of a turnable robot arm (red circled enlargement). The master node is statically placed at a fixed distance in front of the sensor node. (**b**) Receive Signal Strength over spatial angles for different distances (42 cm and 168 cm) and with additional interference of non-moving and moving human tissue (HT). Antenna (cf. PCB in Figure 1) is aligned with 180${}^{\circ }$.

The technical evaluation measurements include recordings of RSSI at an increasing distance with varying transmission power output in a laboratory environment, where the antennas of both sender (SN) and receiver (MN) have been aligned accordingly. As expected according to theoretical considerations following the free-space path loss, the RSSI decreases with increasing distance and decreasing transmission power output. Here, also the transmission output power, matching network loss, and antenna gain are included in the measurement results, which results in an overall offset of approximately −30dBm. In a second experiment, the RSSI was recorded from different spatial angles in a free-space environment, where the sensor node was placed on a turnable end-effector of a robot arm (Figure 10). During the measurement, the sensor node was rotated by 360${}^{\circ }$ in the x-y-plane. The receive signal quality was recorded by the master node that was placed at varying distances (Figure 10b).

Additionally, measurements with static and moving human tissue within the direct signal path were performed. An additional signal attenuation of approximately 5–15 dBm occurs with human tissue interference. For moving human tissue, the RSSI pattern shows inconsistent behavior with both increased and reduced signal attenuation compared to the non-moving measurements. For loss-less data transfer, a minimum of −78dBm RSSI was determined. At 10dBm transmission output power, a maximum of 2.2% PER was found. The proposed sensor system can, therefore, be applied in telehealth sensor systems with human tissue interference.

Finally, we investigated the long-term measurements with regard to the transmission performance of the proposed setup in an everyday-life scenario. Different healthy subjects were asked to wear the measurement setup (sensor nodes at the left wrist, in the right pocket (thigh), at the chest and as an insole (ankle), the master node at the right wrist) in their everyday office life. In addition to the actual sensor data, we additionally recorded the RSSI and the number of packages received by the master node to calculate the PER. The overall results are given in Table 3. The performance evaluation yielded satisfying results with an average PER of 5.45% on average and 7.38% during gait. We performed a correlation analysis between the RSSI and PER values from every single measurement and sensing location, to investigate the relationship between successful packet transmissions and the received signal strength. The correlation analysis yielded an overall correlation coefficient of $\rho_{\mathrm{raw}}=0.778$. The correlation analysis of the averaged performance parameters from Table 3 even resulted in a correlation coefficient of $\rho_{\mathrm{av}}=0.993$. Please note, that we used the absolute RSSI values to correlate a decreasing radio signal strength with an increasing PER tendency. We additionally identified the gait phases within the long-term measurements using the algorithm described in Section 2.6 and calculated both RSSI and PER for each sensor location. The correlation analysis yielded correlation coefficients of $\rho_{\mathrm{gait},\mathrm{raw}}=0.154$ and $\rho_{\mathrm{gait},\mathrm{av}}=0.986$, respectively.

### 3.2. Synchronization Performance

Several long-term measurements were conducted to evaluate the timing performance and long-term stability of the RF communication implementation. The devices under test were placed in a standard technical laboratory environment during the long-term recordings. The SNs were placed around the MN with a distance of about 20cm to ensure optimal transmission performance. We recorded the master timestamp ${\left.T_{\mathrm{MN}}\right|}_{\mathrm{sync}}$ received by the SN, the local slave timestamp ${\left.T_{\mathrm{SN}}\right|}_{\mathrm{sync}}$, and the processing delay $\Delta T_{\mathrm{proc}}$ that are used to compensate for the static timer offset due to different times of initialization and for linear timer drifts due to component tolerances (cf. Section 2.2).

We recorded data of 20 devices under test during the long-term measurements of approximately 1h. Two of the devices showed unsatisfying timing performance yielding many missed synchronization beacons and, thus, non-synchronized timestamps. Subsequently, we separated the test measurements into two groups to further analyze the results: passed timing performance test (18 devices) and failed timing performance test (2 devices). We obtained different indicators to quantify the timing performance of every single device based on the time differences of master and sensor clocks. Therefore, we first calculated the deviation between the sensor clock and the master clock

(11) $$\Delta T_{\mathrm{MN},\mathrm{SN}}\left[k\right]={\left.T_{\mathrm{MN}}\right|}_{\mathrm{sync}}\left[k\right]-{\left.T_{\mathrm{SN}}\right|}_{\mathrm{sync}}\left[k\right]$$

and approximated this time series by a second-order polynomial fit

(12) $$\Delta T_{\mathrm{MN},\mathrm{SN}}\left[k\right]\overset{!}{=}p_{2}{\left(\frac{k}{F_{s}}\right)}^{2}+p_{1}\;\frac{k}{F_{s}}+p_{0}.$$

Based on this approximation, we further analyzed the key characteristics of the oscillator deviations. The results are given in Table 4 on average (mean) for the group of devices that passed the test and for the device with the worst result. Please note that the two devices that failed the test were excluded from the averaging. The divergence $\frac{\Delta T}{s}$ is given by the linear slope $p_{1}$ from Equation (12). Additionally, we calculated the average of the absolute divergence $\left|p_{1}\right|$ for all devices. The temporal linearity of the oscillator misalignment is characterized by the ratio of $\left|\frac{p_{2}}{p_{1}}\right|$. We determined the root mean squares error with regard to the linear fit ${\mathrm{RMSE}}_{\mathrm{lin}}$ to identify short-time irregularities. Finally, we calculated the divergence of the adjusted timestamps ${}^{\mathrm{MN}}T_{\mathrm{SN}}$ to prove the correct operation of the adaption formula in Equation (2). As can be seen, the absolute divergence after adjusting the local clock is significantly below 1μs/s and, therefore, the system can be used for synchronized multi-sensor gait analysis.

### 3.3. Qualitative GRF Measurement

The GRF can be obtained from the FSR sensor data and contains information about the gait stability, gait control strategy, or pathological gait patterns. For example, the position of the Center of Pressure (CoP) and its derivatives are key characteristics in terms of gait stability. It can be deduced from the 2D plantar GRF that is reconstructed by interpolating between the single sensor locations and recalculating the local foot pressure for the specific foot geometry. Further analysis of GRF-related gait quality indicators is not part of this contribution. Instead, we focus on a qualitative validation of the GRF signal morphology. The quality of the signal morphology has a high impact on the detection of HS and TO events using the proposed algorithm. Of course, the morphology individually differs due to foot size, footwear, and contact pressure. Therefore, the insole-based FSR signal is more sensitive to individual gait behavior as compared to the force plates.

**Figure 11:**
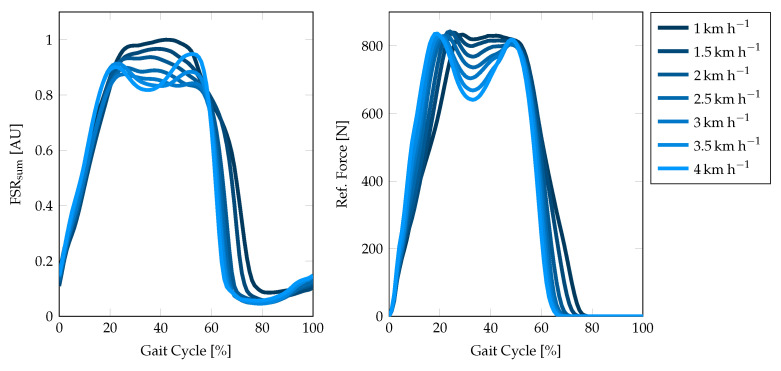
Exemplary comparison of the summed-up FSR signal and the force plate measurement during one walking trial of subject 1 from 1–4 $\mathrm{km}\;h^{-1}$. The graphs show the mean morphology over all gait cycles at one particular speed. The gait cycles have been separated by the HS events detected by the proposed algorithm and the gait analysis software of the treadmill, respectively.

To validate the reproducibility of GRF by the FSR insole, we conducted a clinical trial study with 10 healthy, young participants, who volunteered in a treadmill walking trial. The subjects walked on the treadmill for 7 min per trial at increasing speeds from 1–4 $\mathrm{km}\;h^{-1}$. Each speed step was kept constant for one minute and increased by $0.5\;\mathrm{km}\;h^{-1}$ in between by the trial advisor. The treadmill included integrated force sensors underneath the walking plane, which was used to generate the reference data. The force data from the treadmill can be integrated over the 2D foot area to obtain the vertical GRF of the corresponding gait cycle. Gait cycles within the reference force can be easily detected by thresholding the signal with the smallest possible threshold since the force plate yields zero force during the swing phase. The recorded FSR signal is separated using the algorithm presented in Section 2.7. To analyze the qualitative reproducibility, we averaged over all gait cycles at one speed step for both summed-up FSR and reference force signal. A visual comparison of the FSR measurement and the reference force is depicted in Figure 11.

We additionally calculated the coefficient of variation (CoV), which is defined as the standard deviation in relation to the mean value of a quantity, for the individual stride morphologies of both FSR and reference force plate data to give a measure of variability for the corresponding measurement principle. It should be noted that human gait, of course, inherits a certain amount of stride-to-stride variability. Therefore, the CoV in this case is not a measure of precision for the corresponding technical system. The CoV rather needs to be compared to the reference outcome. Secondly, the swing phase does not contribute to the CoV for the reference system since the effective output is zero when the foot does not have ground contact. As can be seen in Table 5, the CoV of the FSR insole data on average is approximately 40% higher compared to the reference force plate data.

### 3.4. Speed-Related Gait Parameters

The capability of the IMU sensor nodes have been intensively investigated before [38,39] and is, therefore, not in the scope of this contribution. Instead, we focus on the speed-related gait parameters, that we can additionally obtain from the GRF measurements. As key characteristic parameters of human gait, we extract the total number of strides, the stride time, and the stance time from the summed-up FSR signal.

**Figure 12:**
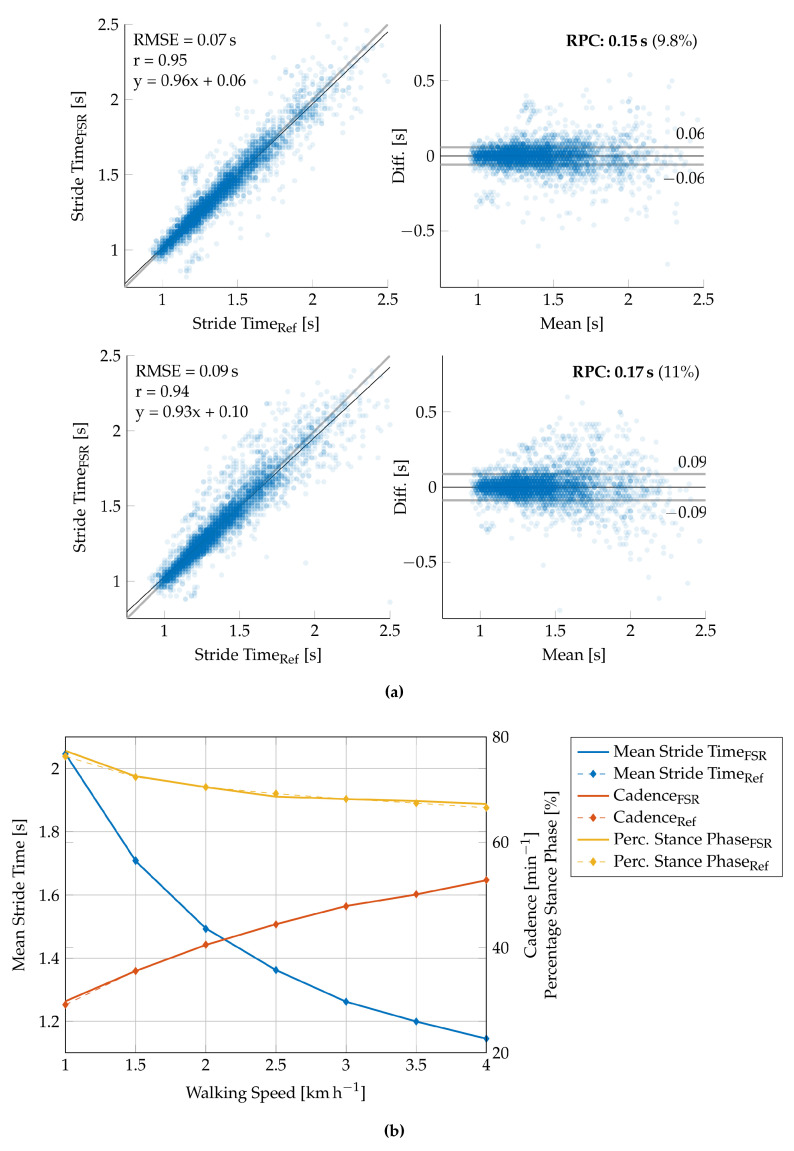
Validation results of the speed-related gait parameters. (**a**) Correlation (**left**) and Bland–Altman (**right**) plot for the HS-based stride time extraction (**above**) and the TO-based stride time extraction (**below**) for all strides detected during walking trials. (**b**) Graphical representation of the overall extraction performance with regard to the calculation of the mean stride time, the cadence, and the percentage stance phase (cf. Table 6).

The stride time is defined as the temporal distance between two successive ground contacts of the same foot. The exact stride time can, therefore, be extracted by any periodically recurring feature in the FSR signal, such as the HS or TO events. Since HS and TO are the two most dominant featured in the FSR signal, we calculated the stride time from both HS and TO and compared it to the reference treadmill data. The correlation and Bland–Altman plots for the HS-based and TO-based stride time extraction are given in Figure 12a. Since HS-based stride time extraction provided more reliable results, we decided to use the HS events for any further parameter calculation. The stride time is thus calculated by

(13) $$\mathrm{ST}\left[l\right]={\left.T\right|}_{{\mathrm{HS}}_{l+1}}-{\left.T\right|}_{{\mathrm{HS}}_{l}}$$

and averaged over a period of one minute to calculate the mean stride time.

The number of strides (NoS) is given by the sum of detected HS events. Concerning the conducted gait study trial, we counted the number of strides in each speed interval, which yields a second important gait parameter: the cadence (strides per minute). We calculated the detection accuracy to give a quantitative measure of the overall stride detection

(14) $$\mathrm{Accuracy}=1-\frac{\left|{\mathrm{NoS}}_{\mathrm{Ref}}-{\mathrm{NoS}}_{\mathrm{FSR}}\right|}{{\mathrm{NoS}}_{\mathrm{Ref}}}.$$

The percentage stance time (PST) indicates the temporal percentage of the stance time in relation to the entire gait cycle (stance time + swing time of one leg) and is calculated by

(15) $$\mathrm{PST}\left[l\right]=\frac{{\left.T\right|}_{{\mathrm{TO}}_{l}}-{\left.T\right|}_{{\mathrm{HS}}_{l}}}{{\left.T\right|}_{{\mathrm{HS}}_{l+1}}-{\left.T\right|}_{{\mathrm{HS}}_{l}}}=\frac{{\left.T\right|}_{{\mathrm{TO}}_{l}}-{\left.T\right|}_{{\mathrm{HS}}_{l}}}{\mathrm{ST}[l]}.$$

Both the mean stride time and the percentage stance time can be validated using the RMSE

(16) $$\mathrm{RMSE}=\sqrt{\sum_{l=1}^{N}\frac{{\left(y_{\mathrm{FSR}}\left[l\right]-y_{\mathrm{Ref}}\left[l\right]\right)}^{2}}{N}},$$

where $y_{\mathrm{FSR}}$ is the respective measure to be validated against $y_{\mathrm{Ref}}$ and *N* equals the number of observations.

The gait sequences were extracted from the overall recording time by applying the gait phase detection from Section 2.6. All measures were subsequently extracted from the summed-up FSR signal according to Equations (13)–(16). The extracted gait parameters are given in Table 6. Additionally, the results of the validation of the speed-related gait parameters are depicted in Figure 12b.

## 4. Discussion

Within this contribution, we introduced the recently redesigned version 3.0 of the IPANEMA BSN based on an 868MHz communication interface. The current version was implemented for the specific application in gait analysis. Therefore, we integrated two different kind of sensor nodes: an IMU node and a sensorized insole for GRF measurement. We proposed a sensor setup to unobtrusively obtain movement data in long-term measurements.

Our results show that the concept of an 868MHz communication interface for a BSN is generally applicable in a measurement setup for gait analysis in long-term data. The averaged RSSI values of approximately −62.5dBm for both the entire data set and gait phases only (cf. Table 3) correspond to the technical evaluation of the transmission performance (approximately −75 to −45dBm, cf. Figure 10b). As expected, the PER increased during the real-life application in the long-term measurements compared to the laboratory measurements (2.2% versus 5.5–7.4%). During gait phases, we also found an increased PER, which can be explained due to additional movement artifacts. Nevertheless, it should be noted that we identified erroneous packets by a simple cyclic redundancy check (CRC) and, thus, the PER equals the packet loss rate. Minor bit errors are currently not corrected, but will be part of future work to reduce the PER. Nevertheless, we showed in Section 3.4 and in [39], respectively, that gait parameter extraction is not drastically affected by missing data points. The correlation between RSSI and PER interestingly decreases during gait phases ($\rho_{\mathrm{gait},\mathrm{raw}}=0.154$ versus $\rho_{\mathrm{raw}}=0.778$). This can be explained by highly dynamic changes of the received signal strength during the gait cycles, which can cause a comparatively high PER, while the average RSSI suggests a stable transmission performance. Please note that transmissions with very low signal strengths are not detected by the receiving master node, which means that theoretically low RSSIs can not be considered for the averaged RSSI calculation. Based on these findings, we consider to adaptively adjust the transmitter output power during the gait phases using a model-predictive approach by characterizing the transmission channel of each individual sensor node. This would not only improve the PER, but also the operating time of the system.

According to Table 1, the maximum operating time of the system due to battery limitations was determined to be about 12h. This corresponds to the currently implemented, maximum timestamp of the local BSN clock (cf. Section 2.2. During the timing evaluation measurements, we found two of the tested devices (10%) that showed large oscillator deviations and, thus, did not function appropriately. Since the deviation of these devices was approximately 50ms/s, the synchronization beacon will be missed every second time and transmission collisions are not guaranteed to be avoided by the TDMA. Additionally, the power consumption of these devices is excessively higher, since once a synchronization beacon is missed, their RF core remains active until the next re-synchronization beacon is received. For these reasons, devices that failed the timing test (absolute deviation $\Delta T_{\mathrm{MN},\mathrm{SN}}>1\;\mathrm{ms}/s$) were excluded from further consideration. The polynomial fit of the absolute deviation of each individual sensor clock yielded high linearity for all devices during the long-term measurements. Therefore, additional nonlinear time-invariant influences on the system need not be expected. Both the mean deviation and mean of the absolute deviation of the devices that passed the test corresponded to the tolerances of the oscillator. Short-time fluctuations of the clock precision were found to be approximately 0.1ms, which is below the global sampling period of $T_{s}=20\;\mathrm{ms}$ of the current BSN system. Thus, the setup satisfies the requirements for a gait analysis application in long-term measurements. In case further different sensor modalities are required, the evaluation of the timing performance must be revised with regard to the required sampling rate. As expected, the absolute divergence of the sensor clock is significantly below the global clock tick of 10μs and is therefore negligible.

A further indicator for validating a system for monitoring and analyzing gait in everyday life is the range of recorded sensor signals with respect to the configured measurement range given in Table 2. During the walking trials in the motion laboratory, we extracted maximum acceleration ranges of approximately [±3.5g] for the thigh sensor, [±1.9g] for the chest sensor, and [±2g] for the wrist sensor. For walking speeds covered by the proposed study, the chosen range is, therefore, sufficient. In the case that higher values are expected, for example from an accelerometer located at the ankle or foot, the range can be extended by software. Similar values were also observed during the long-term measurements. Temporary instances of senor saturation could be attributed to short-term, high-energy events that were not associated with the gait phases. In addition to the IMU, the BPS is also an important component of the mobility sensor nodes at the thigh, the wrist, and the chest. In the current sensor configuration, the BPS provides an effective precision of approximately ±10.9cm at sea level due to the measurement noise. Nevertheless it should be noted that the barometric pressure measurement is highly sensitive to minor pressure fluctuations, such as closing or opening of doors or windows, and changes in temperature. During the long-term measurements, we could observe that significant changes in altitude, such as floor elevating and stairs walking could be simply detected within the BPS data, whereas the detection of minor changes requires additional signal processing. First approaches to detect falls or even sit-to-stand transitions yielded promising results, while taking into account the additionally recorded motion data. This topic will, therefore, be subject of future investigations. In the current sensor setup, the signal noise of the pressure sensor equals a standard deviation of the altitude estimate of approximately 10.9cm at sea level (cf. Table 2). In addition, the pressure measurement is highly sensitive to temperature changes and minor pressure fluctuations, such as the opening or closing of doors or windows.

The FSR insole is used to calculate important speed-related gait parameters, such as the total number of strides, the cadence, the stride time, and the percentage stance phase. GRF-based analysis is known to provide reliable results for these parameters and therefore often used in movement laboratories. The applicability to a wearable long-term monitoring system is evaluated in this contribution. The results of the validation measurements in the movement laboratory are given in Table 6 and Figure 12, respectively. First, we investigated the stride detection performance of both HS and TO-based by comparing the extracted stride time to the reference stride time (Figure 12a). As the correlation plots and Bland–Altman plots show, the HS-based stride time extraction slightly outperforms the TO-based calculation (RMSE 0.07s versus 0.09s). These results may be explained by the foot anatomy and resulting pressure distributions and additional forces due to contact pressure of the shoe. We assume that the heel sensor (${\mathrm{FSR}}_{1}$, Figure 4a) yields a more precise and better defined signal contribution as compared to the ball of the foot and the toes (${\mathrm{FSR}}_{2}$–${\mathrm{FSR}}_{4}$). Therefore, we decided to use the HS-based event detection for further parameter extraction. Furthermore, the reference data of the force plates (cf. Figure 11) suggest the conclusion that besides HS and TO further gait phase parameters such as mid stance can be extracted from the GRF signal. Nevertheless, this is a challenging task with regard to FSR-based measurements since the signal morphology is often corrupted by additional motion artifacts of the insole.

The qualitative validation of the GRF measurements demonstrated that the proposed sensor setup satisfies the requirements of an unobtrusive gait analysis system. Although the signal morphology differs between the implemented insole and the reference force plate data (cf. Figure 11), it can be concluded that four FSR sensors located at the proposed landmarks provide sufficient information for further gait parameter extraction. Similar setups with only FSR sensors integrated also reported the reliability of this arrangement [27,42]. Since the system makes no claim to an exact calculation of the CoP lines or even pressure prints for the detection of foot malpositioning, an extension of the FSR insole is currently not necessary. However, an appropriate calibration routine of the FSR sensors to map the measured resistance to an effective force or pressure, can improve the FSR-based parameter extraction. Nevertheless, we proved the basic concept with the presented FSR sensor arrangement to extract gait parameters. Since the FSR sensors cover the most important points that have ground contact during stance phase, the summed-up FSR signal can be assumed to majorly reflect the GRF morphology (cf. Figure 11). Since the detection of HS and TO events is based on the summed-up FSR signal, no matter which sensor is the main contributor at which time of the gait cycle, it can be assumed that this also applies to pathological gait patterns such as they may occur in equinus foot. Within this contribution, we presented a study with healthy young participants without any diagnosed gait impairments, such that we were not able to prove this hypothesis. A more intensive investigation of the FSR sensor technology regarding the ability to reflect pathological gait patterns, the dynamic repeatability and long-term stability for the development of a calibration routine will therefore be part of the future work.

The number of strides reflect the sum of the detected HS events. The cadence can be calculated from the number of strides per minute. Table 6 shows that the proposed stride detection algorithm is able to detect most strides with an overall accuracy of 99.6±0.8%, which is comparable to the recently published setup of an FSR insole for step counting purposes [27]. Here, the authors conducted a trial with indoor and outdoor walking at self-selected (approximately $5.2\;\mathrm{km}\;h^{-1}$) and maximal speed (approximately $6.3\;\mathrm{km}\;h^{-1}$) and achieved an accuracy of 99.5% and 99.6%, respectively. It should be noted that we intentionally included slow walking speeds 1–4 $\mathrm{km}\;h^{-1}$ in our study due to the target application in the monitoring of movement disorders, which are often accompanied by reduced walking speeds. At very slow walking speeds (1–1.5 $\mathrm{km}\;h^{-1}$) our proposed system provided worst results due to the increased amount of movement artifacts during the transitions of swing and stance phase and the swing phase itself. The increased error in gait parameter extraction at very slow walking speeds is, therefore, due to incorrect gait segmentation. As can be seen in Figure 12a, the error variance of the individual stride times most likely increases with decreasing walking speed. Nevertheless, averaging over a certain period of time can compensate for this effect to a certain extent since deviations in the stride time determination will have the opposite effect on the subsequent gait cycle (cf. Table 6). At higher walking speeds of 2–4 $\mathrm{km}\;h^{-1}$, the system yielded satisfying results (99.9%).

The mean stride time can be either calculated by the reciprocal cadence or the mean over the temporal distance between two HS events. In this case, we have calculated the stride-to-stride interval and averaged over the recording period of one minute. In contrast to the direct comparison of the stride times (cf. Figure 12a), the mean stride time yields a very high correlation with the reference data featuring an RMSE of approximately 5ms. An accurate calculation of the mean stride time is of course directly connected to the accuracy of the stride detection. Therefore, these results correspond to the formerly discussed performance of the stride detection algorithm.

As can be seen in Figure 11, the summed-up FSR signal highly correlates with the force plate measurements regarding the falling and rising edges due to the HS and TO events. However, the determination of the exact time of HS and TO still is a challenging task due to movement artifacts of the shoe contact pressure during the swing phase. The percentage of the stance phase in relation to the total stride time varies by approximately 10% at the given walking speeds of 1–4 $\mathrm{km}\;h^{-1}$ (cf. Table 6). This change was equally observed in both FSR and reference data. The FSR-based calculation of the PST resulted in an RMSE of approximately 2.3%, which is acceptable for a wearable gait monitoring system. A significant difference was found in the standard deviation of the PST calculation, which corresponds to the assumption that FSR-based HS and TO detection are less accurate compared to the force plate measurements. Since the FSR-based PST extraction has proven to be reliable, we will expand the system in the future to include a second insole that is worn in the opposite shoe. This will provide the possibility to extract additional gait parameters, such as the single and the double support time, to identify the load response and the pre-swing phase or even to obtain an approximation of the CoP lines.

The cadence, the mean stride time and the percentage stance phase are of course important parameters to observe the progression of the locomotor system or superordinated movement disorders, which initially requires a reliable event detection. Nevertheless, the variability of different gait parameters, such as the stride time variability, contains valuable information about gait stability [39]. The robust stride detection thus fulfills an important requirement of a long-term monitoring system, but also an exact temporal determination of the stride event is of high interest. Therefore, the focus of the future work is to improve the stride-to-stride interval extraction, which not only concerns the algorithmic approaches but also a robust transmission and an accurate time synchronization. On the sensor side, future developments include the addition of an IMU on the insole for a more precise detection of HS and TO by fusing this information with the FSR data. The inclusion of information from the other IMU sensors at the wrist, chest, and thigh is an important part of the future algorithmic work to further improve the accuracy of the gait parameter extraction.

## 5. Conclusions

In this contribution, we presented the recently developed version 3.0 of the IPANEMA BSN based on an 868MHz communication interface. We evaluated the transmission performance and the synchronization protocol with regard to an application in a wearable long-term monitoring system for unobtrusive gait analysis. We demonstrated the general functionality of the system within technical laboratory measurements and during long-term measurements in everyday life activities in an office environment. We proposed two different kinds of sensing modalities, IMU motion sensors and FSR-based insoles for GRF measurements, and validated the insoles in a study trial against reference data obtained in a movement laboratory. Therefore, we presented an algorithm to identify the HS and TO events in the summed-up FSR signal. We extracted three different clinically relevant gait parameters, the number of strides, or the cadence, respectively, the mean stride time and the percentage stance time. The system proved an overall satisfying performance for an application in a long-term monitoring setup to gain information of clinically relevant parameters in the patient’s everyday life.

## Figures and Tables

**Table 1:** Comparison of main characteristics of the IPANEMA v2.5 and v3.0 mainboard.

Feature	IPANEMA v2.5 (2011)	IPANEMA v3.0 (2019)
Controller	MSP430F1611	CC1310
Architecture	16-Bit RISC	ARM Cortex-M3
RF Transceiver	CC1101	integrated
Frequency Band	433 MHz	868 MHz/915 MHz
max. TX Power	10 dBm	14 dBm
TX Current *	29.2 mA	13.4 mA
RX Current	15.7 mA	5.5 mA
Battery	330 mAh	230 mAh
Operating Time **	ca. 4 h	ca. 12 h
max. Data Rate	500 kBit/s	4 MBit/s
Sensitivity	−116 dBm	−124 dBm

* At transmission output power of 10 dBm, ** mainboard equipped with an inertial measurement sensor.

**Table 2:** Characteristic parameters of the IMU sensor nodes implemented for the BSN.

Parameter	Accelerometer	Gyroscope	Pressure Sensor
Range	±4g	$2000^{\circ }/s$	300…1100hPa
Resolution	14bits	16bits	17bits
Noise	$150\;\mu g/\sqrt{\mathrm{Hz}}$	$0.014^{\circ }/s/\sqrt{\mathrm{Hz}}$	1.3Pa
Sampling Rate	100Hz	100Hz	125Hz

**Table 3:** Transmission performance during long-term measurements from unsupervised everyday office life activities. The calculation of RSSI and Packet Error Rate (PER) was performed for the entire data set (all) and gait phases only (gait) to specifically investigate the transmission behavior during gait.

Data	Parameter	Wrist	Thigh	Chest	Ankle	Averaged
All	RSSI [dBm]	−62.80	−63.41	−56.95	−68.32	−62.87
	PER [%]	5.42	6.36	1.16	8.87	5.45
Gait	RSSI [dBm]	−65.07	−60.11	−57.10	−67.32	−62.40
	PER [%]	9.34	4.76	3.75	11.68	7.38

**Table 4:** Results of the synchronization evaluation.

Indicator	Average	Worst Case
	Passed Test	
Divergence [μs/s]	−11.69	−−
Abs. Divergence [μs/s]	18.63	46.445.59
Linearity	$2.907\times 10^{-6}$	$3.29\times 10^{-8}$
${\mathrm{RMSE}}_{\mathrm{lin}}$ [s]	$1.081\times 10^{-4}$	0.025
Abs. Adj. Divergence [μs/s]	$20.532\times 10^{-3}$	$7.539\times 10^{-3}$

**Table 5:** Coefficient of variation [] for the mean FSR morphology compared to the reference morphology obtained from the treadmill force plates.

Data	Walking Speed [km h${}^{-1}$]						
	1.0	1.5	2.0	2.5	3.0	3.5	4.0
${\mathrm{CoV}}_{\mathrm{FSR}}$	0.126	0.108	0.095	0.091	0.099	0.091	0.108
${\mathrm{CoV}}_{\mathrm{Ref}}$	0.112	0.084	0.071	0.065	0.061	0.061	0.065

**Table 6:** Results of the extraction of speed-related gait parameters: the number of strides, the mean stride time, and the percentage of the stance phase. Values are given in mean±standarddeviation.

	Number of Strides/Cadence			Mean Stride Time			Percentage Stance Phase		
Speed [km h${}^{-1}$]	FSR [min${}^{-1}$]	Ref. [min${}^{-1}$]	Accuracy [%]	FSR [s]	Ref. [s]	RMSE [ms]	FSR [%]	Ref. [%]	RMSE [%]
1.0	29.8±4.25	29.1±4.15	97.8±1.62	2.05±0.31	2.05±0.32	22.36	77.36±3.96	76.30±2.99	2.76
1.5	35.5±3.73	35.5±3.66	99.7±0.58	1.71±0.21	1.71±0.21	2.80	72.53±3.22	72.37±1.79	2.47
2.0	40.5±3.46	40.5±3.44	99.9±0.38	1.49±0.15	1.49±0.15	7.93	70.45±3.02	70.45±1.53	2.53
2.5	44.5±3.55	44.4±3.56	99.9±0.34	1.36±0.12	1.36±0.12	0.35	68.65±2.10	69.22±1.35	1.70
3.0	47.9±3.73	47.9±3.77	99.9±0.33	1.26±0.11	1.26±0.11	0.40	68.18±2.19	68.20±1.21	1.99
3.5	50.1±3.09	50.1±3.05	99.9±0.31	1.20±0.08	1.20±0.08	0.77	67.81±2.70	67.40±1.31	2.52
4.0	52.8±3.38	52.9±3.35	99.9±0.29	1.14±0.08	1.14±0.08	0.58	67.24±2.00	66.55±1.27	2.10
Average	43.0±8.5	42.9±8.6	99.6±1.0	1.46±0.3	1.46±0.3	5.027	70.32±4.4	70.07±3.6	2.296
